# Primary Care Provider Perspectives on Expanded Genomic Screening in Children

**DOI:** 10.21203/rs.3.rs-7012425/v1

**Published:** 2025-09-09

**Authors:** Elizabeth Kathleen Branch, Megan C Roberts, Margaret Waltz, Neal A. deJong, Laura V. Milko, Ann Katherine M. Foreman, Kimberly Foss, Stefanija Giric, Marcella H. Boynton, Jonathan S. Berg, Samantha Schilling

**Affiliations:** UNC School of Medicine Chapel Hill; UNC Eshelman School of Pharmacy; UNC School of Medicine Chapel Hill; UNC School of Medicine Chapel Hill; UNC School of Medicine Chapel Hill; UNC School of Medicine Chapel Hill; UNC School of Medicine Chapel Hill; UNC School of Medicine Chapel Hill; UNC North Carolina Translational and Clinical Sciences (NC TraCS) Institute; UNC School of Medicine Chapel Hill; UNC School of Medicine Chapel Hill

## Abstract

**Objective(s):**

Expanding pediatric genomic screening beyond current newborn screening presents both opportunities and challenges to primary care providers. We are developing a novel paradigm called Age-Based Genomic Screening (ABGS), which will incorporate targeted genomic sequencing for select, highly actionable genetic conditions into routine care at relevant time-points throughout childhood. We surveyed pediatric primary care providers in North Carolina to identify potential ABGS implementation determinants and strategies to address them.

**Study design:**

We disseminated an electronic survey to family medicine and pediatric primary care clinicians. Survey items were modeled on constructs previously identified as important to genomic medicine and assessed perceived utility, benefits, barriers, and facilitators of implementing targeted genomic screening in pediatric primary care. Data were analyzed using descriptive statistics and content analysis, as appropriate.

**Results:**

A total of 93 individuals completed the survey. Over 85% of respondents agreed that genomic screening was important and impactful in their patient care but about 30% lacked confidence in their ability to implement it in their practice. The most cited benefits of the ABGS program were related to readiness for implementation and the evidence, strength, and quality of the intervention. The most concerning barriers included cost for patients and available resources, with 87% and 75% of respondents having extreme or moderate concern for these barriers, respectively.

**Conclusion(s):**

Our findings have implications both for the design of the ABGS pilot program and directions for future research in genomic implementation. In particular, the blueprint for the pilot program must include specific plans for ensuring primary care providers have the time and resources available for shared decision making with their patients about engaging in genomic screening.

## INTRODUCTION

Applications of genomic testing in health care are rapidly proliferating. Availability of faster, more cost effective, and accurate testing – and growing public acceptance of its value – has resulted in efforts to incorporate genomic testing into public health screening programs, primarily focused during the newborn period and adulthood.^1–4^ A growing body of literature assesses implementation in these settings^5–7^ and the importance of engaging key participants (parents and providers) to provide information on strategies for successful implementation.^8–11^

Researchers have developed several frameworks that can be applied in diverse settings to characterize implementation determinants used in genomic screening efforts. One example is the IGNITE Network’s Common Measures Working Group framework, which utilized the Consolidated Framework for Implementation Research (CFIR) to prioritize 10 constructs for the implementation of genomic medicine in clinical settings.^12,13^ In addition to characterizing common barriers to implementation of genomic screening, implementation scientists have also identified approaches for supporting public health outcomes that equally benefit multiple groups, including involving patients and other community partners to identify patient-centered approaches.^14–16^ Equally important to identifying implementation determinants is addressing them through implementation strategies. The Expert Recommendations for Implementing Change (ERIC) study used concept mapping to assess the feasibility and importance of various implementation strategies used to overcome implementation barriers, including those encountered in genomic medicine.^17^

These frameworks, among others from implementation science, have helped to identify and address challenges of implementing ethical, patient-centered genomic screening in adult healthcare settings, as well as during the expansion of newborn screening.^18^ Common challenges reported by providers participating in clinical genomic research include the enrollment and education of diverse populations, smooth integration of new processes into existing workflows, connecting patients to accessible follow-up genetics care, and a perceived lack of self-efficacy among providers to deliver genomic interventions.^6,19,20^ Frequently identified implementation facilitators include the adaptability of the intervention, collaboration between the research and clinical teams, and the relative priority of the intervention particularly among enthusiastic clinical staff.^6,19,20^

Although many implementation determinants from adult and newborn genomic screening programs have been identified in prior studies, less is understood about potential barriers and opportunities for novel approaches to genomic screening throughout childhood and adolescence. Age Based Genomic Screening (ABGS) is a novel program under development that aims to incorporate targeted genomic sequencing during routine pediatric well child visits throughout childhood for a select number of highly actionable genetic conditions.^21,22^ Although the details of ABGS are still in development, the program will likely comprise of 3 genomic testing panels offered to children at specific well child checks. Each panel will consist of genetic conditions with age of onset soon after the age of testing and actionable management options to ameliorate or prevent disease.^21,22^ The feasibility of the program will be tested in a pilot study designed through collaboration with researchers, pediatric primary care providers, specialty genetics providers, and community members. The goal of this study was to identify ABGS implementation determinants and potential strategies to address them.

## METHODS

### Overview

We conducted a survey of pediatric primary care providers across North Carolina (NC) regarding their perceived barriers and benefits to implementing routine, age-based genomic screening into primary care. The study protocol was reviewed by the Institutional Review Board of the Office of Human Research Ethics of the University of North Carolina and was determined to be exempt from Human Subjects Research Review.

### Participants

Clinicians and practice managers employed in pediatric and family medicine clinics enrolled in the North Carolina Network Consortium (NCNC) were invited to participate. NCNC is a diverse, statewide consortium of providers, academic institutions, and patients whose mission is to address pressing questions related to the delivery of primary care health services and the management of primary care problems.

### Enrollment Procedures

Study enrollment took place from October 2023 to February 2024. Email invitations were sent to practice managers of clinics enrolled in NCNC. Contacted practice managers were asked to forward the study invitation to all physicians and practice managers within their clinic. Up to two reminder email invitations were sent to non-responding clinics. The email invitation included a brief description of the study, a flyer providing additional study details, and a link to the survey in Qualtrics. Participants received a $20 gift card after completing the survey.

### Survey Instrument

A custom survey was developed for this study, included in supplementary files. Survey items were informed by high-priority constructs identified by the IGNITE Network Common Measures Working Group from CFIR and results from the ERIC study.^12,17^ Items assessed perceived barriers and benefits for implementing targeted genomic screening in pediatric primary care and potential strategies to overcome them. Feedback from pilot testing of the survey was incorporated prior to initiating data collection.

**Figure 1:**
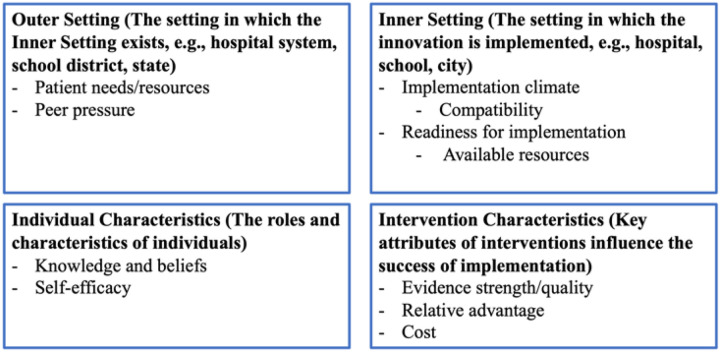
CFIR domains and constructs represented by survey questions

Respondents indicated their general perceptions of genomic screening through relative agreement with five statements reflecting constructs from the Characteristic of the Individual CFIR domain, with statements investigating specific constructs: Knowledge and Beliefs (three items) and Self-efficacy (two items). A brief paragraph explaining the goals of ABGS was provided followed by questions asking respondents to rate the degree of importance (high, medium, low, not at all) or concern (extremely, moderately, slightly, not at all) of potential benefits and barriers of the program, respectively. Nine potential benefits were included and represented CFIR domains Intervention, Inner Setting, and Outer Setting. Nine potential barriers were included and represented CFIR domains Intervention, Inner Setting, and Individuals’ characteristics (Fig. 1). Participants were invited to share additional insights regarding perceived barriers and benefits of genomic screening via free text.

**Figure 2:**
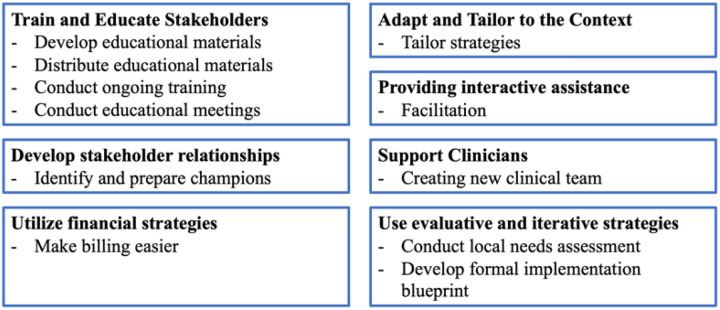
ERIC clusters and strategies represented by survey questions

Respondents were asked to rank four aspects of the ABGS program based on which would be most likely to influence the feasibility of implementing ABGS in their clinic. Eight implementation strategies were then presented, and respondents were asked to rate the degree to which each strategy may be helpful in the implementation of the AGBS program in the clinical setting on a Likert-type scale (very, somewhat, not very, or not at all helpful). The implementation strategies related to ERIC clusters Change Infrastructure, Train and Educate Stakeholders, Support Clinicians, Utilize Financial Strategies, Use Evaluative and Iterative Strategies, and Adapt and Tailor to Context (Fig. 2).^17^ Participants were invited to share additional insights regarding implementation strategies via free text.

The following demographic information was collected from respondents: practice county, respondent role (practice manager, clinical care coordinator, physician, nurse practitioner, certified nurse midwife, physician assistant, other), age, race, ethnicity, and gender. Respondents that identified as a physician, nurse practitioner, physician assistant, or certified nurse midwife received additional questions regarding years of primary care practice and experience with abnormal newborn screening results.

### Analysis

Quantitative data were analyzed using descriptive statistics. To analyze open text responses, we developed an *a priori* codebook using the IGNITE constructs and ERIC strategies on which the survey was based. Two coders independently coded the open text responses using direct content analysis.^23,24^ Coding was compared and discrepancies were brought to the larger team for review. We then organized the coded material by code and reviewed the material to identify overarching themes.^25^ Finally, through triangulation, we compared the closed- and open-ended survey responses, identifying where responses converged and complemented each other.^26^

## RESULTS

### Study Participants

An invitation to participate was emailed to 61 clinics. A total of 93 individuals from 55 clinics completed the survey and were included in the analysis. Based on the way NCNC operates, study teams are not informed if the practice manager forwards the study email to their clinical team, or how many members comprise each clinical team at the time the study invitation is sent. Because of this, we were not able to calculate an accurate response rate for survey participants. Most respondents identified as women (62%) and as White (73%) (Table 1). Nearly 75% of respondents were physicians.

### General Perceptions of Genomic screening

Over 85% of respondents agreed that information generated by genomic screening is important for patient care and that genomic screening is relevant to their current clinical practice (Table 2). Fewer respondents were confident in discussing genomic screening with patients and using the results of genomic screening for patient care, with 32% and 41% disagreeing with each statement, respectively.

### Perceived Benefits of ABGS

The potential benefits to genomic screening in the pediatric context rated as most important included providing patients access to medical experts when screening identifies a rare genetic condition and the opportunity to identify rare diseases prior to symptom onset with 86% and 84% of respondents ranking each benefit as highly important, respectively (Table 3). Improving outcomes for children on a population level and the potential to reduce disparities in recognition of genetic conditions were also viewed as key benefits with 79% and 71% of respondents ranking them highly important, respectively. In contrast, only 27% of respondents rated placing their practice at the forefront of innovations in pediatric medicine as a benefit of high importance.

In the free text responses, respondents included comments that fell within CFIR domains of Inner setting, Outer setting, Individual characteristics, and Intervention characteristics. Several free text responses mirrored the benefits identified in the quantitative results, including increasing patient access to medical experts and identifying rare diseases prior to symptom onset. For instance, one respondent said: “I agree that ABGS is needed and necessary in our clinics and am dedicated to work together to ensure that we are able to assist patients who may be interested.”Another respondent shared, “More and more parents ask us about genetic issues in their families. I think this [ABGS] could be a very helpful thing for our patients.” One respondent highlighted the importance of prevention: “ABGS could decrease long term costs by engaging in prevention.” Notably, there was some divergence among respondents who commented on the potential of ABGS to reduce health disparities. While one respondent commented, “Caring equally for underserved and rural and urban patients is a key benefit of the proposed program,” another respondent questioned if the proposed program would achieve this benefit: “Screening or offering to all patients is only one facet of reducing disparities in recognition. If you want to reduce the recognition disparity this should be more robust.”

### Perceived Barriers of ABGS

Approximately 87% of respondents were moderately or extremely concerned about the potential financial cost of ABGS for patients and their families (Table 4). Access to timely consultation with a genetic specialist was another commonly perceived barrier, with 75% of respondents identifying this to be of extreme or moderate concern. Respondents were less concerned about potential barriers related to lack of evidence, patient interest, or patient need with 68%, 75%, and 85% noting slight or no concern about these potential barriers, respectively.

Free text responses describing barriers fell within CFIR domains of Outer setting, Inner setting, Individuals’ characteristics, and Intervention characteristics. Similar to the quantitative results, concerns over time were apparent in the free text responses, as exemplified in the following: “Our well child visits are already overburdened; preventive care is not reimbursed adequately enough to allow us to spend more than 15 minutes with our patients and we already have more things to cover than we have time for so it’s hard to imagine adding another thing to counsel, test, and communicate about.” Another respondent shared, “We already have a lot of other things ongoing in our practice (all of which are ‘valuable’). Fitting in ‘one more thing’ is the problem.” Another wrote, “Well child checks have become increasingly busy, and time is limited.” How the ABGS program will impact populations differentially was also referenced as an important barrier: “Implementing this testing assumes everyone has equal access, no cost burden in an anti-racist system. Sickle cell is the best example of a disease easily identified but poorly treated because of discrimination,” and “Patients with limited numeracy and literacy will face specific challenges to interpretation, as will patients who speak languages other than English.” One respondent brought up a concern not represented in our survey questions related to the long-term cost to the patient: “One barrier is what if the patient has future health/life insurance denied due to the screening results?”

### ABGS- Feasibility

About one third of respondents (34%) ranked specimen collection manner as the most important ABGS feasibility priority, followed by comprehensiveness of supporting materials and resources (27%). The number of different time points at which ABGS would be offered and the ability to receive reimbursement for time spent counseling families about genomic screening were each ranked as the highest feasibility priority by 14% of respondents.

### ABGS- Implementation Strategies

The implementation strategy reported as being the most helpful was giving providers succinct information materials about ABGS with 82% of respondents identifying it as very helpful (Table 5). A direct point of contact to the research team for potentially interested families to reach out to about ABGS was also perceived to be important with 77% of respondents ranking it as very helpful.

The ERIC clusters most represented in the free text responses related to implementation strategies were Train and Educate Stakeholders, Support Clinicians, and Utilize Financial Strategies. Open-ended responses further highlighted the importance of developing and distributing educational materials for patients and families: “Provide a short video explaining the service, testing, and implications that patients are required to watch prior to testing.” Several other open-ended responses emphasized the importance of supporting clinicians: “YOU MUST remove the work burden from providers. They should not be the genetic counselor. I think this should be done as a separate visit immediately before or after care guided by separate personnel to discuss this with the patient,” and “Ideally, having the results automatically route to an individual/provider/practice with experience with these genetic screenings as many providers in my clinic are not familiar with the result interpretation or follow-up. Or, having an on-call individual available to e-consult or staff message on epic.” Finally, one respondent stressed the importance of having a detailed implementation plan: “The key is in the details —what is being screened, how often, method (finger stick vs. venous vs. swab), potential impact (incidence, false negatives, preventive health impact, cost benefit) etc.”

## DISCUSSION

Our findings indicate enthusiasm and interest for expanded genomic screening among pediatric primary care providers across NC. Respondents showed particular interest in the potential benefits of the ABGS program to help patients access care before symptoms arise and to help reduce disparities within healthcare. Interestingly, the potential to increase healthcare disparities was also a concerning barrier to the implementation of ABGS, along with time and expertise limitations within primary care to effectively implement the program. Implementation strategies that focused on addressing financial (cost for patients) and operation (timely access to consultation, provider time limitations within visits) barriers were prioritized by respondents as essential to successful implementation of ABGS.

These results reinforce implementation determinants previously described in the literature, including the enrollment and education of diverse populations and the smooth integration of new processes into existing workflows.^6,14,19^ While mirrored in previous literature describing programs targeting newborn and adult populations, the concern of time limitations among providers was particularly emphasized in our study, perhaps representing the unique challenge of adequately covering a wide variety of topics within a pediatric well-child visit and the additional burden of parental education.^6,20^ The unique structure of ABGS spanning multiple well child care visits posits this survey to provide novel data on provider perspectives on implementation determinants and strategies of expanded screening in a longitudinal setting.

Our findings have implications for the ABGS pilot program’s design and other genomic medicine practices. For example, in addition to being prioritized in our study, in a study examining the implementation of pharmacogenetics testing in various clinic settings the CFIR constructs of patient needs and resources, knowledge and beliefs of individuals, and evidence strength and quality of the intervention, emerged as top priorities.^27^ Similar to our study, the challenges associated with the role of primary care providers in delivering genetic results has been studied in the implementation of other genomic medicine practices. Studies have explored primary care providers’ views on discussing with patients the genetic risk of common chronic diseases and actionable genomic findings from a research biobank.^28,29^ Much like our results, these studies indicate a widespread belief that genetic test results are important to delivery of high-quality care, but also that primary care providers lack confidence to accurately deliver such results.^28,29^ These sentiments were mirrored in our survey responses regarding providers’ trust in the relevance and importance of programs such as ABGS, while simultaneously lacking confidence in their ability to discuss genetic findings with their patients. The implementation strategies rated as most helpful by survey respondents (providing succinct informational materials and a direct point of contact within the research study), while relevant to ABGS, may also be helpful in the implementation of other genomic screening programs.

The successful implementation of the ABGS program hinges on our commitment to address the survey findings described in this study in the design of our planned pilot. The structure of a pre-implementation survey of barriers and benefits followed by the creation of a pilot blueprint has similarly been used in the implementation of other genomic medicine practices, including the adoption of a family health history risk assessment with great success.^30^ For ABGS specifically, to address the implementation determinants we identified, we need to include specific plans for ensuring primary care providers have the time and resources available for shared decision making with their patients regarding engaging in screening. In the case of a positive result, the pilot program must provide guided navigation of our complex healthcare system including timely access to follow-up care.

Perhaps most importantly, the design, implementation, and critical evaluation of the ABGS pilot program must address the acknowledged potential of ABGS to reduce or perpetuate disparities within the healthcare system referenced by many survey respondents. Various components of ABGS underway in parallel to the initial survey described in this paper are laying a framework for anticipating and addressing the health disparities associated with the implementation of genomic medicine initiatives. The project includes a Community Engagement and Education Committee focused on recruiting diverse members of NC communities to share their concerns and endorsements of all stages of the ABGS project. As part of this committee, we have created a Community Advisory Board consisting of a diverse group of parents that are members of the local community who bring unique and critical perspectives to the pilot study’s design, focusing on the experience of patients and their families.^11^

Next steps in the design and development of the ABGS program include several iterations of feedback planned for the pilot protocol including individual interviews with providers, practice managers, and patients and families, observation of clinic workflow, and feedback sessions discussing implementation of the pilot into existing clinic processes. The implementation of the pilot will include the collection of data that can be analyzed to understand what went well and what needs to be addressed in future expansion of the ABGS program. These data will be analyzed within the framework provided by this study to identify benefits and barriers that were and were not addressed with the pilot implementation plan. There is much to be learned from the future stages of this project with implications for expanded genetic screening and other genomic medicine initiatives.

Our study has limitations. Although the NCNC consortium is large and diverse, it is possible that respondents from clinics not a part of this group might have provided a novel perspective with respect to genetic screening in the pediatric context. Additionally, because all of our respondents are from NC, the generalizability of our results to other states and contexts is unknown. The results may represent regional bias in healthcare practice, policy, and provider perspectives influenced by multiple factors such as the current status of newborn screening and Medicaid expansion in NC. Due to the limitations of NCNC, were unable to measure how many of the practice managers forwarded the survey invitation to their clinics, or the number of clinicians actively practicing within each clinic, thus limiting our ability to calculate the survey response rate. Furthermore, as with all surveys, there is likely sampling bias with providers who are more interested in genomics screening being more likely to respond, thus skewing the results. Not all respondents provided free text comments; however, the goal of the free text responses was not to reach thematic saturation but rather understand additional barriers, benefits, and strategies not represented in survey items. In addition, our sample size was relatively small although represented geographic diversity across the state. Our study was not powered to evaluate responses by region or demographic characteristic of respondents which could have been informative to future implementation. Despite these limitations, the results will be sufficient to support future ABGS program development and implementation as described.

## CONCLUSIONS

Our findings have implications for the ABGS pilot program’s design and directions for future research in genomic implementation. In particular, the pilot program must address providers’ concerns regarding the resources needed to equitably and sustainably expand genetic screening in primary care settings. The potential for the program to perpetuate health disparities and overwhelm care providers must be top of mind with every design decision regarding the pilot and the evaluation of its success following completion. The comparison of these pre-implementation survey results with a critical evaluation of the pilot’s ability to address providers concerns will inform future implementation of genomic medicine initiatives.

## Supplementary Files

This is a list of supplementary files associated with this preprint. Click to download.

- ABGSTier1QualtricsSurvey.docx

## Figures and Tables

**Table 1:** Demographic characteristics.

Characteristic		n(%) or M ± SD
Gender*		
	Man	33 (36)
	Woman	58 (62)
	Transgender	1 (1)
Race/Ethnicity*		
	White	68 (73)
	Asian	16 (17)
	Black or African American	7 (8)
	Hispanic/Latino	4 (4)
	Native Hawaiian or Other Pacific Islander	1 (1)
	Prefer not to answer	3 (3)
NC Regions		
	Coastal	8 (9)
	Eastern	55 (59)
	Central	17 (18)
	Northern	9 (10)
	Western	0 (0)
	Multiple counties	2 (2)
	Missing	2 (2)
Role in practice		
	Practice manager	4 (4)
	Physician	69 (74)
	Nurse practitioner	3 (3)
	PA	1 (1)
	Other	12 (13)
	Certified nurse midwife	2 (2)
	Missing	2 (2)
Years practicing primary care		
	0–5 years	20 (22)
	6–10 years	14 (15)
	11–15 years	8 (9)
	More than 15 years	33 (36)
	Missing	18 (19)
Number of times need to follow up on abnormal result for metabolic screen		
	Never	11 (12)
	1–2 times	24 (26)
	3–5 times	16 (17)
	6 + times	24 (26)
	Missing	18 (19)

* Select all that apply

**Table 2:** General Perceptions of Genomic screening in Pediatric Primary Care

CFIR Domain-Construct		Strongly Disagree	Somewhat Disagree	Somewhat Agree	Strongly Agree	Missing
	General Perceptions of Genomic screening	n (%)	n (%)	n (%)	n (%)	n (%)
Individual characteristics-Knowledge and beliefs	The information generated by genomic screening is important for patient care.	1 (1)	2 (2)	33 (36)	55 (59)	2 (2)
	I believe that genomic screening is relevant to my current clinical practice.	0 (0)	9 (10)	38 (41)	44 (47)	2 (2)
	Genomic screening will improve my ability to care for patients.	1 (1)	10 (11)	40 (43)	40 (43)	2 (2)
Individual characteristics-Self efficacy	I am comfortable talking about genomic screening with my patients and their families.	13 (14)	17 (18)	42 (45)	19 (20)	2 (2)
	I am confident in my ability to use the results of genomic screening with my patients.	11 (12)	27 (29)	39 (42)	13 (14)	3 (3)

**Table 3:** Perceived Benefits of ABGS

CFIR Domain	CFIR Construct		High Importance	Medium Importance	Low or No Importance	Missing
		ABGS Benefits	n (%)	n (%)	n (%)	n (%)
Inner	Readiness for implementation-available resources	Providing my patients access to medical experts for follow up when screening identifies a rare genetic condition.	80 (86)	11 (12)	0 (0)	2 (2)
Intervention	Evidence strength/quality	Opportunity to identify rare diseases prior to symptom onset that can lead to prompt clinical intervention.	78 (84)	11 (12)	2 (2)	2 (2)
Outer	Patient needs/resources	Improving outcomes for pediatric patients on a population level.	73 (79)	15 (16)	2 (2)	3 (3)
Intervention	Relative advantage	Potential to reduce disparities in recognition of genetic conditions by screening all patients.	66 (71)	23 (25)	2 (2)	2 (2)
Intervention	Relative advantage	Increasing access to advanced screening for my patients.	53 (57)	31 (33)	7 (8)	2 (2)
Intervention	Relative advantage	Potential to eliminate diagnostic evaluations for some patients with rare genetic diseases.	42 (45)	43 (46)	6 (7)	2 (2)
Outer	Patient needs/resources	Help my patients and their families learn more about how genetics affects health.	42 (45)	38 (41)	11 (12)	2 (2)
Intervention	Relative advantage	Help me be more involved in the care of my patients with genetic diseases.	46 (50)	28 (30)	17 (18)	2 (2)
Outer	Peer pressure	Placing my practice at the forefront of innovations in pediatric medicine.	25 (27)	27 (29)	38 (41)	3 (3)

**Table 4:** Perceived Barriers of ABGS

CFIR Domain	CFIR Construct		Extremely Concerned	Moderately Concerned	Slightly or Not at all Concerned	Missing
		ABGS Barriers	n (%)	n (%)	n (%)	n (%)
Intervention	Cost	It could lead to significant financial cost for our patients and their families.	44 (47)	37 (40)	10 (11)	2 (2)
Inner	Readiness for implementation-available resources	Access to timely consultation with genetic specialists is inadequate.	42 (45)	28 (30)	21 (23)	2 (2)
Individuals	Knowledge/beliefs	It could lead to increased anxiety for our patients and their families.	33 (36)	26 (28)	32 (34)	2 (2)
Inner	Readiness for implementation-available resources	It would take too much provider time during well visits.	26 (28)	35 (38)	30 (32)	2 (2)
Individuals	Self-efficacy	Our clinic providers would have limited confidence discussing it.	11 (12)	43 (46)	37 (40)	2 (2)
Inner	Implementation climate-compatibility	It will be difficult to incorporate into our existing clinic workflow.	16 (17)	28 (30)	47 (51)	2 (2)
Intervention	Evidence strength/quality	The evidence is not strong enough to support it.	6 (7)	21 (23)	63 (68)	3 (3)
Individuals	Knowledge/beliefs	Our patients would not be interested in it.	1 (1)	19 (20)	70 (75)	3 (2)
Individuals	Knowledge/beliefs	Our patients do not need it.	2 (2)	10 (11)	79 (85)	2 (2)

**Table 5.** Perceived helpfulness of implementation strategies.

ERIC Cluster	ERIC Strategy	Implementation Strategies	Very helpful	Somewhat helpful	Not very/at all helpful	Missing
			n (%)	n (%)	n (%)	n (%)
Train and Educate Stakeholders | Adapt and tailor to the context	Distribute educational materials + Tailor strategies	Low literacy and bilingual information materials about ABGS for patients (e.g., dot phrase for After Visit Summary about purpose of screening, possible outcomes, and next steps).	68 (73)	21 (23)	2 (2)	2 (2)
Support Clinicians	Creating new clinical teams	A direct point of contact to the research team for potentially interested families to reach out to about ABGS.	72 (77)	18 (19)	1 (1)	2 (2)
Providing interactive assistance	Facilitation	Providing hands-on implementation support during the pilot.	58 (62)	28 (30)	5 (5)	2 (2)
Train and Education Stakeholders	Develop/distribute educational materials	Succinct information materials about ABGS for providers.	76 (82)	13 (14)	2 (2)	2 (2)
Train and Education Stakeholders	Conduct ongoing training/educational meetings	Educational Inservice about genetic conditions and genetic testing/screening for your practice.	67 (72)	19 (20)	5 (5)	2 (2)
Use evaluative and iterative strategies	Conduct local needs assessment	Completion of a brief assessment to determine what needs to be addressed to incorporate ABGS in your clinic.	56 (60)	30 (32)	5 (5)	2 (2)
Utilize financial strategies	Make billing easier	A guide for coding and billing, including counseling time and dot phrases for documentation.	71 (76)	17 (18)	3 (3)	2 (2)
Use evaluative and iterative strategies| Adapt and tailor to context	Develop a formal implementation blueprint| Tailor strategies	A written plan outlining the roles, responsibilities, and processes for ABGS developed by our team and adapted with your input to fit your practice needs.	64 (69)	22 (24)	5 (5)	2 (2)
Develop stakeholder interrelationships	Identify and prepare champions	Identification and support of provider champions for ABGS in your practice.	49 (53)	34 (37)	8 (9)	2 (2)

## Data Availability

The datasets used and/or analysed during the current study are available from the corresponding author on reasonable request.
